# Emergence of IncHI2 Plasmids With *Mobilized Colistin Resistance* (*mcr*)-*9* Gene in ESBL-Producing, Multidrug-Resistant *Salmonella* Typhimurium and Its Monophasic Variant ST34 From Food-Producing Animals in Italy

**DOI:** 10.3389/fmicb.2021.705230

**Published:** 2021-07-16

**Authors:** Elena Lavinia Diaconu, Patricia Alba, Fabiola Feltrin, Paola Di Matteo, Manuela Iurescia, Eleonora Chelli, Valentina Donati, Ilaria Marani, Angelo Giacomi, Alessia Franco, Virginia Carfora

**Affiliations:** National Reference Laboratory for Antimicrobial Resistance, General Diagnostics Department, Istituto Zooprofilattico Sperimentale del Lazio e della Toscana “M. Aleandri,” Rome, Italy

**Keywords:** *Salmonella* Typhimurium, monophasic variant, *mcr-9*, whole genome sequencing, long-read sequencing, multidrug resistance, ESBL, IncHI2 plasmid

## Abstract

A collection of 177 genomes of *Salmonella* Typhimurium and its monophasic variant isolated in 2014–2019 from Italian poultry/livestock (*n* = 165) and foodstuff (*n* = 12), previously screened for antimicrobial susceptibility and assigned to ST34 and single-locus variants, were studied in-depth to check the presence of the novel *mcr-9* gene and to investigate their genetic relatedness by whole genome sequencing (WGS). The study of accessory resistance genes revealed the presence of *mcr*-9.1 in 11 ST34 isolates, displaying elevated colistin minimum inhibitory concentration values up to 2 mg/L and also a multidrug-resistant (MDR) profile toward up to seven antimicrobial classes. Five of them were also extended-spectrum beta-lactamases producers (*bla*_*SHV*__–__12_ type), mediated by the corresponding antimicrobial resistance (AMR) accessory genes. All *mcr*-9-positive isolates harbored IncHI2-ST1 plasmids. From the results of the Mash analysis performed on all 177 genomes, the 11 *mcr-9*-positive isolates fell together in the same subcluster and were all closely related. This subcluster included also two *mcr*-9-negative isolates, and other eight *mcr*-9-negative ST34 isolates were present within the same parental branch. All the 21 isolates within this branch presented an IncHI2/2A plasmid and a similar MDR gene pattern. In three representative *mcr*-9-positive isolates, *mcr-9* was demonstrated to be located on different IncHI2/IncHI2A large-size (∼277–297 kb) plasmids, using a combined Illumina–Oxford Nanopore WGS approach. These plasmids were also compared by BLAST analysis with publicly available IncHI2 plasmid sequences harboring *mcr*-9. In our plasmids, *mcr*-9 was located in a ∼30-kb region lacking different genetic elements of the typical core structure of *mcr*-9 cassettes. In this region were also identified different genes involved in heavy metal metabolism. Our results underline how genomics and WGS-based surveillance are increasingly indispensable to achieve better insights into the genetic environment and features of plasmid-mediated AMR, as in the case of such IncHI2 plasmids harboring other MDR genes beside *mcr*-9, that can be transferred horizontally also to other major *Salmonella* serovars spreading along the food chain.

## Introduction

Salmonellosis, among the most important foodborne zoonoses worldwide, is the second most commonly reported gastrointestinal infection in humans after campylobacteriosis in the European Union/European Economic Area, mainly caused by the consumption of contaminated food (European Food Safety Authority [EFSA] and European Centre for Disease Prevention and Control [ECDC], 2021). In Europe, among all confirmed salmonellosis cases, the three most commonly reported *Salmonella* serovars in 2019 were *Salmonella* Enteritidis (50.3%), *Salmonella* Typhimurium (11.9%), and monophasic *Salmonella* Typhimurium (1,4,[5],12:i:-) (8.2%) (European Food Safety Authority [EFSA] and European Centre for Disease Prevention and Control [ECDC], 2021). Sequence types (STs) 19, 34, 313, and 213 represent the most frequently reported STs for *S.* Typhimurium. Starting from the past two decades, the global pandemic *S.* Typhimurium ST34 clone has been increasingly reported, replacing to the “traditional” clone ST19 (Biswas et al., 2019). The efficacy of antimicrobial therapy for salmonellosis may be impaired by the spread of antimicrobial-resistant isolates (Michael and Schwarz, 2016), particularly to last-resort drugs used to treat severe infections in humans.

Colistin is a last-resort antibiotic of the polymyxin family, increasingly used for treating human invasive infections by multidrug-resistant (MDR) or extensively drug-resistant (XDR) Enterobacteriaceae (Poirel et al., 2017). To date, 10 different *mcr* genes have been reported worldwide in Enterobacteriaceae (Wang et al., 2020), with 6 of them (*mcr*-1 to *mcr*-5 and *mcr*-9) identified so far in *Salmonella enterica* (Lima et al., 2019); MDR monophasic *S.* Typhimurium ST34 harboring *mcr* genes (*mcr*-1, *mcr*-3, and *mcr*-5) has also been widely reported worldwide.

*mcr-9* is the most recent *mcr* homolog (65 and 63% amino acid identity with *mcr*-3 and *mcr*-7, respectively) so far described in *Salmonella* serovars in different countries in samples from humans (Carroll et al., 2019; Pan et al., 2020; Wang et al., 2021), horses (Elbediwi et al., 2021), livestock (Borowiak et al., 2020; Leite et al., 2020), food (Borowiak et al., 2020; Cha et al., 2020; Tyson et al., 2020; Wang et al., 2021), and environment (Wang et al., 2021).

*mcr-9* is an inducible plasmid-borne gene mainly associated with the IncHI2 replicon type (Li et al., 2020), encoding an acquired phosphoethanolamine transferase not conferring by itself clinical resistance to colistin. However, subinhibitory concentrations of colistin could induce its expression, which, in *E. coli*, is mediated by a two-component regulatory system encoded by the *qse*C and *qse*B genes, located downstream of *mcr*-9 (Kieffer et al., 2019).

The aims of this work were to (i) reanalyze by whole genome sequencing (WGS) a historical collection of *S.* Typhimurium and its monophasic variant within ST34 (along with its single-locus variants) to screen for the presence of *mcr-9* and to investigate the relatedness of their genomes and (ii) determine the genetic environment of the *mcr*-9 gene and fully reconstruct the *mcr*-9-harboring plasmids by using a combined Illumina–Oxford Nanopore WGS approach.

## Materials and Methods

### *Salmonella* Isolates

A collection of 177 genomes of *S.* Typhimurium (*n* = 39) and its monophasic variant (*n* = 138) assigned to ST34 and single-locus variants by WGS (Supplementary Table 1) was studied in-depth. These isolates were obtained from animal samples (*n* = 165), collected from Italian poultry (*n* = 1 broiler chicken, *n* = 2 laying hens, and *n* = 14 fattening turkeys) and livestock [*n* = 106 fattening pigs and *n* = 42 bovine animals <12 months (veal calves)] and also foodstuff (*n* = 12), in the frame of antimicrobial resistance (AMR)-monitoring activities (according to Decision 2013/652/EU^1^) conducted from 2014 to 2019 by the National Reference Laboratory for Antimicrobial Resistance (NRL-AR) and previously screened for antimicrobial susceptibility.

### Antimicrobial Susceptibility Testing (AST)

AST was performed as minimum inhibitory concentration (MIC) determination by broth microdilution, using the EU consensus 96-well microtiter plates (Trek Diagnostic Systems, Westlake, OH, United States). The results were interpreted according to epidemiological cutoffs (ECOFFs) included in the Annex A of the EU Decision 2013/652/EU, and for sulfamethoxazole the tentative ECOFF of >256 mg/L according to the EURL-AR protocol for antimicrobial susceptibility testing of *Escherichia coli, Salmonella*, and *Campylobacter^2^*. *E. coli* ATCC 25922 was used as quality control strain.

### Illumina Short-Read Sequencing and Bioinformatics Analysis

WGS was first performed using an Illumina platform (MiSeq). DNA extraction, library preparation, trimming, and *de novo* assembly of raw reads were performed according to Alba et al. (2020).

Molecular characterization was performed on all the assembled genomes with the ABRicate tool^3^ using the Genomic Epidemiology (CGE) databases of ResFinder^4^ and PlasmidFinder^5^, and the MLST^6^ and SeqSero2 (Zhang et al., 2019) tools for the detection of the genetic basis of AMR and plasmid replicon types and to confirm the STs and the serotype *in silico*, respectively.

The pMLST 2.0 online version^7^ was also used for pMLST analysis on *mcr*-9-positive isolates and in representative *mcr*-9-negative isolates. The presence of the two-component regulatory system of *mcr*-9 inducible expression encoded by the *qseC* and *qseB* genes (Kieffer et al., 2019), was also searched by BLAST analysis.

In order to determine the genetic relatedness of the whole set of *Salmonella* genomes analyzed, all raw reads from the 177 genomes were also compared and clustered by using the Mash algorithm (Katz et al., 2019).

### Oxford Nanopore Technologies (ONT) Long-Read Sequencing and Bioinformatics Analysis

In order to resolve the complete sequence, fully reconstruct the *mcr*-9-harboring plasmids, and precisely identify and locate *mcr*-9-harboring regions, three selected *mcr*-9-positive (IDs: 19063952, 15060500, and 19093665) and one *mcr*-9-negative (ID: 17021625; one of the most closely related to positive ones selected for comparison purposes) isolates were also sequenced using the nanopore-based MinION device (ONT) with the rapid barcoding kit (SQK-RBK004). A hybrid (Illumina–Oxford Nanopore) assembly was performed using the Unicycler pipeline (Branton et al., 2008) with the default parameters.

The assembly obtained was annotated using the RAST Server (Aziz et al., 2008). Additionally, a manual curation for the obtained annotation was performed, especially for the Insertion Sequences (ISs) by using the ISfinder database (Siguier et al., 2006). The presence of virulence genes was also determined on the obtained plasmid sequences using the VF database^8^.

The *mcr*-9-carrying plasmids obtained were also compared using BLAST (Altschul et al., 1990) with two previously published complete *mcr*-9-carrying plasmid sequences from Enterobacteriacae: (a) an *mcr*-9/*bla*_*VIM*__–__1_-carrying plasmid named p3846_IncHI2_mcr (accession number CP052871) with a size of 293,138 bp, isolated from *Enterobacter cloacae* firstly identified in Italy from a human clinical case (Marchetti et al., 2021); (b) an *mcr*-9/*bla*_*VIM*__–__1_-carrying plasmid named pRH-R27 (accession number LN555650.1) with a size of 299,305 bp from a *Salmonella* infantis isolated from a fattening pig farm in Germany (Falgenhauer et al., 2017).

Graphical representation of the full plasmid comparison, of the *mcr*-9-specific region, and dendrogram was carried out using different tools: BRIG (Alikhan et al., 2011), EasyFig (Sullivan et al., 2011), and iTol (Letunic and Bork, 2021), respectively.

## Results

### AST

The AMR phenotypes of all Salmonella isolates are reported in Supplementary Table 1. As for the 11 *mcr*-9-positive isolates, beside the AMR gene profiles, also the MIC values are reported in detail in Table 1.

**TABLE 1:** Genotypic and phenotypic characterization of the *mcr-*9 and selected *mcr-*1 carrying isolates analyzed by WGS.

						Antimicrobial resistance profile		Plasmid content	
Isolate ID	Serotype *in silico*	Origin	Year of isolation	ENA accession number	ST	Horizontally acquired genes	Phenotypic AST profile (MIC value mg/L)	Plasmid replicons	IncHI2 Plasmid MLST (pMLST)
19015927	I 4,[5],12:i:-	Fattening pigs-cecum	2019	ERS6592624	34	*bla*_*TEM*__–1B_, *aph*(3’)-Ia, *cat*A2, *aac(6′)-*Iaa, ARR3, *dfr*A27, *aad*A16, *sul*1, *mcr*-9, *aac*(3)-IVa, *aph*(4)-Ia, *flo*R, *tet*D, *aph*(3″)-*Ib*, *aph*(6)-Id	CHL(256), GEN(32), TMP(64), TET(128), AMP(128), SMX(2048) COL (2)	IncHI2A, IncHI2, IncQ1	ST1
17037369	I 4,[5],12:i:-	Fattening pigs-cecum	2017	ERS6592625	34	*aac(6′)-*Iaa, *bla*_*SHV*_12, *aac*(3)-IId, *sul*2, *aph*(3″)-Ib, *aph*(6)-Id, *mcr*-9, *bla*_*TEM*__–1B_, *cat*A1, *qnr*S1	CHL(256), GEN(64), TET(64), AMP(128), CIP(0,5), FOT(8), TAZ(16), SMX(2048) COL (1)	IncHI2, IncHI2A	ST1
15049009	I 4,[5],12:i:-	Fattening pigs-cecum	2015	ERS2030266	34	*aac*(6′)IIc, *tet*B, *dfr*A19, *flo*R, *tet*D, *mcr*-9, *sul*1, *aph*(6)-Id, *aac*(6′)-Iaa, *aad*A2	CHL(256), GEN(64), TMP(64), TET(128), AMP(128), SMX(2048) COL (1)	IncHI2, IncHI2A	ST1
15060498	I 4,[5],12:i:-	veal calves production animals-cecum	2015	ERS2030252	34	*bla*_*TEM*__–1B_, *aac*(6′)-Iaa, *flo*R, *tet*B, *sul*2, *tet*D, *mcr*-9, *qnr*A1, *dfr*A19, *aph*(6)-Id, *aad*A2, *sul*1	CHL(128), TMP(64), TET(64), AMP(128), CIP(0,25) SMX(2048) COL (1)	IncHI2, IncHI2A	ST1
15060500°	I 4,[5],12:i:-	veal calves production animals-cecum	2015	ERZ2110487	34	*bla*_*TEM*__–1B_^§^, *aac*(6′)-Iaa, *tet*B, *flo*R^§^, *sul*2^§^, *tet*D^§^, *mcr*-9^§^, *aph*(6)-Id^§^	CHL(128), TET(128), AMP(128), SMX(2048), COL (1)	IncHI2, IncHI2A	ST1
15083030	Typhimurium	Fattening pigs-cecum	2015	ERS2030200		*bla*_*TEM*__–1B_, *aac*(6′)-Iaa, *bla*_*SHV*_12, *tet*B, *mcr*-9, *cat*A1	CHL(256), TET(128), AMP(128)FOT(4), TAZ(16) COL (1)	IncHI2, IncHI2A	ST1
19041082	I 4,[5],12:i:-	Fattening pigs-cecum	2019	ERS6592626	34	*bla*_*TEM*__–1B_, *mcr*-9, *aac*(3)-IVa, *aph*(4)-Ia, *tet*B, *aac*(6′)-IIc, *qnr*B2, *bla*_*SHV*_12, *flo*R, *aph*(3′)-Ia, *sul*2, *tet*C, *aac*(6′)Iaa, *dfr*A19, *cat*A2, *aph*(6)-Id, *aad*A2, *sul*1	CHL(256), GEN(64), TMP(64), TET(128), AMP(128), CIP(0,5), FOT(8),TAZ(16), SMX(2048) COL (2)	IncHI2, IncHI2A, IncI1-I(Gamma)	ST1
19093665°	I 4,[5],12:i:-	Fattening pigs-cecum	2019	ERZ2110531	34	*aac*(6′)-Iaa, *bla*_*TEM*__–1B_^§^, *aac*(3)-IVa, *aph*(4)-Ia, *aac*(6′)-IIc^§^, *sul*1^§^, *bla*_*SHV*_12^§^, *dfr*A19^§^, *flo*R, *tet*D^§^, *mcr*-9^§^, *aph*(6)-Id^§^	CHL(256), GEN(64), TMP(64), TET(128), AMP(128), FOT(8), TAZ(16), SMX(2048) COL (1)	IncHI2, IncHI2A, IncI1-I(Gamma)	ST1
19057303	I 4,[5],12:i:-	veal calves production animals-cecum	2019	ERS6592627	34	*mcr*-9, *qnr*A1, *sul*1, *aph*(6)-Id, *aph*(3″)-Ib, *bla*_*TEM*__–1B_, *aad*A2, *sul*2, *aac*(6′)-Iaa, *tet*B, *aac*(6′)-IIc, *flo*R, *dfr*A19, *te*tD	CHL(256), GEN(64), TMP(64), TET(128), AMP(128), CIP(0,5), SMX(2048) COL (2)	IncHI2, IncHI2A	ST1
15045799	I 4,[5],12:i:-	Fattening pigs-cecum	2015	ERS2030275	34	*aac*(6′)-Iaa, *tet*B, *aac*(6′)-IIc, *dfr*A19, *bla*_*SHV*_12, *tet*D, *mcr*-9, *qnr*A1, *sul*1, *bla*_*TEM*__–1B_, *aph(*6)-Id, *aad*A2	GEN(64), TMP(64), TET(128), AMP(128), CIP(0,5), FOT(4), TAZ(16), SMX(2048) COL (1)	IncHI2, IncHI2A	ST1
19063952°	I 4,[5],12:i:-	Carcase swab from pig	2019	ERZ2110542	34	*aph*(6)-Id^§^, *aad*A2^§^, *aac*(6′)-Iaa, *tet*B, *aac*(6′)-IIc^§^, *dfr*A19^§^, *sul*2^§^, *bla*_*SHV*_12^§^, *tet*D^§^, *mcr*-9^§^, *sul*1^§^, *bla*_*TEM*__–1B_^§^	GEN(64), TMP(64), TET(128), AMP(128), FOT(8), TAZ(16), SMX(2048) COL (2)	IncHI2, IncHI2A	ST1
17021625*	I 4,[5],12:i:-	Fattening pigs-cecum	2017	ERZ2110516	34	*aac*(6′)-Iaa, *tet*A^§^, *mcr*-1.1, *flo*R^§^, *sul*1^§^, *dfr*A1^§^, *tet*M^§^, *aph*(3′)-Ia^§^, *sul*2^§^, *sul*3^§^, *qnr*B19, *bla*_*TEM*__–1B_^§^, *aph*(6)-Id^§^	CHL(256), NAL(64), TMP(64), COL(8), TET(128), AMP(128), CIP(2), TGC(2), SMX(2048) COL (8)	Col(pHAD28), IncHI2, IncHI2A	ST4

AST, Antimicrobial susceptibility test results associated with AMR genetic background; ENA, European Nucleotide Archive, ST = sequence type following the scheme of Enterobase (https://enterobase.warwick.ac.uk/); AMR, antimicrobial resistance; AMP, ampicillin; CHL, chloramphenicol; CIP, ciprofloxacin; FOT, cefotaxime; GEN, gentamicin; SMX, sulfamethoxazole; TAZ, ceftazidime; TET, tetracycline; TMP, trimethoprim; TGC, tigecycline; *mcr-9-negative isolate, long reads sequenced, °mcr-9-positive isolate, long reads sequenced, ^§^ AMR gene located on IncHI2 plasmid revealed by the hybrid (Illumina–Oxford Nanopore) approach.

For isolates reported in Table 1, the MIC values of highest priority critically important antimicrobials (HPCIAs) such as colistin, cefotaxime, ceftazidime, and ciprofloxacin were interpreted also according to European Committee on Antimicrobial Susceptibility Testing (EUCAST)^9^ clinical breakpoints. In synthesis, out of 11 isolates, 6 showed microbiological and clinical resistance to cefotaxime (FOT) and ceftazidime (TAZ) (MIC range 4–8 mg/L for FOT and 16 mg/L for TAZ). Five showed microbiological resistance (MIC range 0.25–0.5 mg/L), and one also clinical resistance (MIC = 2 mg/L) to ciprofloxacin (CIP). The 11 *mcr*-9-positive isolates displayed elevated colistin MIC values: 4 of them had a MIC of 2 mg/L, on the epidemiological cutoff and clinical breakpoint, and 7 had a MIC of 1 mg/L.

### Illumina Short-Read Sequencing and Bioinformatics Analysis

**FIGURE 1:**
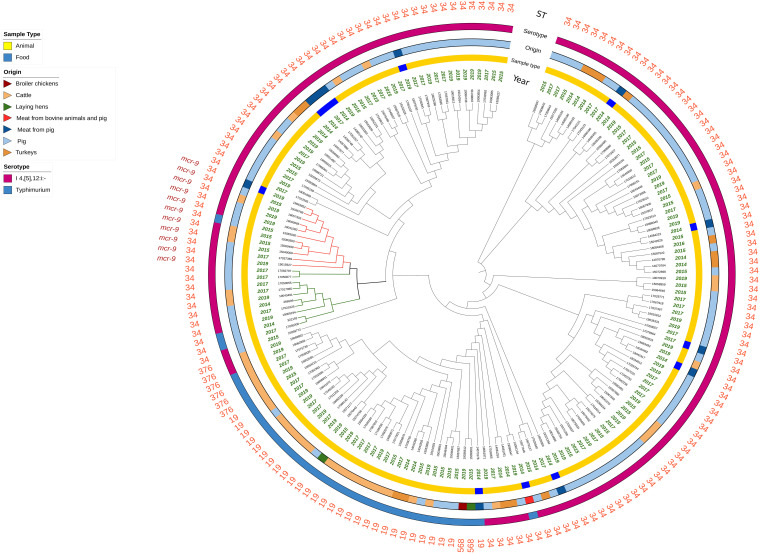
Circular representation of the Mash analysis of the 177 *Salmonella* genomes analyzed. Branch length is not proportional to the genetic distance. The red branches indicate the mcr-9-positive isolates harboring IncHI2 plasmids, and the green branches indicate the mcr-9-negative isolates harboring IncHI2 plasmids.

Most of the *S*. Typhimurium and monophasic variant isolates were confirmed as belonging to ST34 (*n* = 140), while *n* = 37 to single-locus variants ST19 (*n* = 29), ST568 (*n* = 2), and ST376 (*n* = 6) (Figure 1 and Supplementary Table 1). The study of accessory resistance genes revealed the presence of *mcr*-9.1 in 11 ST34 isolates (1 *S.* Typhimurium and 10 monophasic variant) from the cecal content of seven pigs, three veal calves, and from a pig carcase swab (Table 1 and Supplementary Table 1), but none of them displayed phenotypic resistance to colistin (7 isolates displayed MIC values of 1 mg/L and 4 isolates of 2 mg/L). Details of genomic characteristics analyzed by WGS of the 11 *mcr*-9-positive isolates are reported in Table 1. These isolates displayed an MDR gene profile toward up to seven antimicrobial classes (aminoglycosides, beta-lactams, trimethoprim, tetracyclines, sulfamethoxazole (fluoro)quinolones, and phenicols), mediated by AMR accessory genes. In particular, beyond the presence of the *mcr*-9 gene, 9 of the 11 isolates presented the *aac(6′)-Iaa*, *aph(6)-Id*, *bla*_*TEM*__–1B_ gene pattern associated, respectively, with amikacin/tobramycin, streptomycin, and β-lactam resistance. Eight of them also harbored gentamicin resistance genes as *aac*(3)-IV and/or *aac*(3)-IId and/or *aac*(6′)-IIc combined with *sul*1 and/or *sul*2 (seven isolates), *tet*(B) and/or *tet*(D) (six isolates), *dfrA19* or *dfrA27* (six isolates), the extended-spectrum beta-lactamases (ESBL) gene *bla*_*SHV*__–__12_ (five isolates), *flo*R and/or *catA1* and/or *catA2* (five isolates), *qnrA1* or *qnrB2* or *qnrS1* (four isolates) genes, encoding, respectively, sulfamethoxazole, tetracycline, trimethoprim, all beta-lactams, phenicol, and fluoroquinolone resistance. The abovementioned AMR gene profiles were confirmed by the corresponding AMR phenotypes (Table 1). No *mcr*-9-positive isolates presented known chromosomal point mutations associated with AMR phenotypes, and all tested negative for the presence of *qseC* and *qseB* regulatory genes. All *mcr*-9-positive isolates harbored an IncHI2/2A, two an IncI1 and one an IncQ replicon type. Moreover, the pMLST analysis revealed that all of them harbored an IncHI2-ST1 plasmid, while one representative *mcr*-9-negative isolate harbored an IncHI2-ST4 replicon (Table 1).

The results of the Mash analysis are represented in Figure 1. In the Mash tree, three main clusters were identified, with two of them consisting of 7 and 29 ST34 monophasic variant isolates, and the third one, being the most complex, grouped all the remaining isolates in several subclusters. No clear clustering based on year of sampling, sample type, or origin of the isolates was observed. The 11 *mcr-9*-positive isolates fell together in one of the subclusters within the most complex cluster and were all closely related (marked in red, Figure 1). This subcluster included also two *mcr*-9-negative isolates (17050877 and 17082797 marked in green, Figure 1). Additionally, other eight *mcr*-9-negative ST34 isolates were present within the same parental branch (marked in green, Figure 1).

**FIGURE 2:**
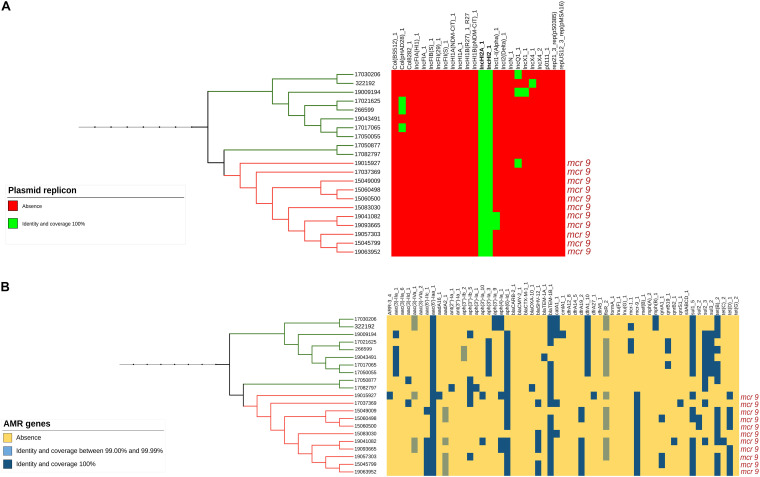
**(A)** Presence or absence of plasmid replicons identified by Mash analysis in the mcr-9-positive and related mcr-9-negative isolates. **(B)** Presence or absence of AMR genes identified by Mash analysis in the mcr-9-positive and related mcr-9-negative isolates.

Only the 21 isolates within this branch (both the 11 *mcr*-9 positive and the 10 negative ones) presented an IncHI2/2A plasmid (Figure 2A and Supplementary Figure 1A) and a similar MDR gene pattern, with three of them being also positive for *mcr*-1.1, associated with colistin resistance (Figure 2B and Supplementary Figure 1B).

### ONT Long-Read Sequencing and Bioinformatics Analysis

Three representative *mcr*-9-positive and one *mcr*-9-negative isolates (Table 1 and Supplementary Table 1) were selected to be sequenced with ONT. From the hybrid (Illumina–Oxford Nanopore) assembly approach, the three plasmids containing *mcr-9* were resolved. *mcr-9* was demonstrated to be located on three different IncHI2/IncHI2A plasmids, named pMOL952, pMOL665, and pMOL500, with sizes of 297, 486, 291, 132, and 277,503 bp, respectively. The *mcr-9*-negative isolate also contained an IncHI2/IncHI2A plasmid, named pMOL625, with a size of 283, 630 bp.

**FIGURE 3:**
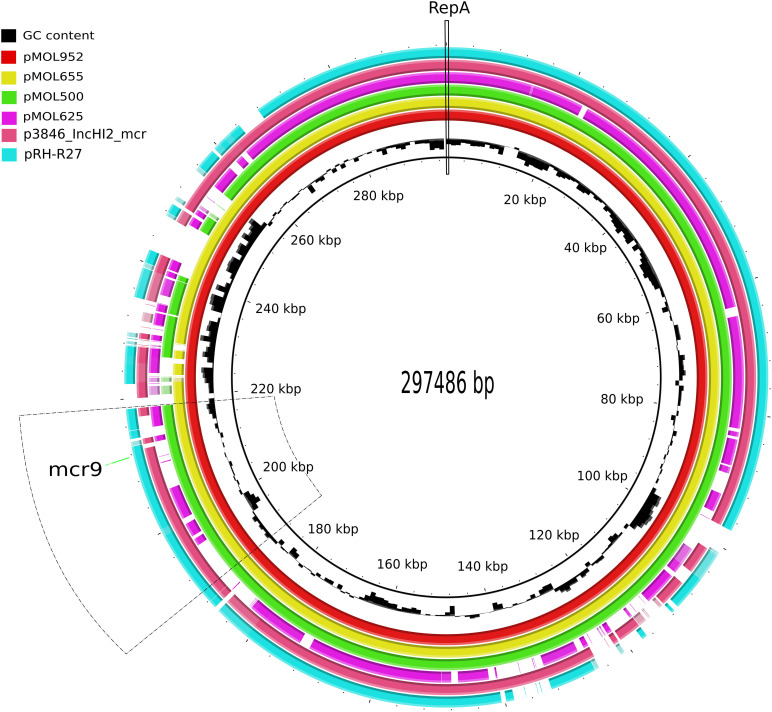
Graphical comparison of the complete sequence of pMOL-952 (red; ERZ2110542), pMOL-665 (yellow; ERZ2110531), pMOL-500 (green; ERZ2110487), pMOL625 (fuchsia; ERZ2110516), p3846_IncHI2 (old pink; CP052871), and pRH-R27 (blue marine; LN555650.1). The repA gene is indicated with a continuous line. The region including mcr-9 is indicated with a dotted line.

Annotation of plasmid sequences identified four main genetic regions in the four IncHI2/IncHI2A plasmids. The plasmid backbone (located in the region 1..40.000 nucleotides (nt); ∼13% of the total plasmid size) included a replication region containing the *rep*A gene encoding a replication initiation protein, a stability region, and the conjugative and transfer genes (Figure 3).

Comparison of our three *mcr*-9-carrying plasmids revealed that with a coverage range of 94–98%, they shared an identity of 99.9%, while the *mcr*-9-negative pMOL625 presented a lower coverage range (71–75%) and an identity of 98.7% with the abovementioned plasmids. Besides *mcr*-9, all the four IncHI2 plasmids harbored the majority of the AMR genes detected encoding resistance to all beta-lactams, aminoglycosides, trimethoprim, tetracyclines, sulfamethoxazole, and phenicols (Table 1). In particular, pMOL-952 and pMOL-665 shared a variable region (240,020..250,837 nt; Figure 3) of a class 1 integron carrying several AMR genes and *qacE*Δ*1* (quaternary ammonium compound resistance gene). The studied plasmids also harbored genes involved in the metabolism of heavy metals as arsenic, nickel, and mercury. In this regard, a mercury-resistance (mer) operon (101,550..105,526 nt; Figure 3) was shared by the *mcr*-9-positive pMOL-952, pMOL-665, and pMOL-500 plasmids and was absent in the *mcr*-9-negative pMOL625.

No virulence genes were detected in the four IncHI2 plasmids.

**FIGURE 4:**
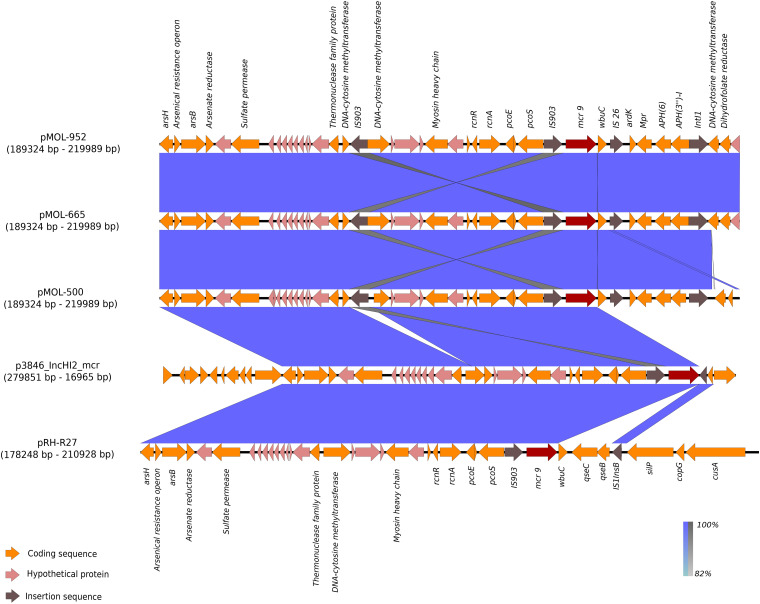
BLAST graphical output of the region where mcr-9 is located in pMOL-952 (ERZ2110542), pMOL-665 (ERZ2110531), pMOL-500 (ERZ2110487), p3846_IncHI2 (CP052871), and pRH-R27 (LN555650.1). Red arrows: mcr-9 gene, orange arrows: Genes encoding functional proteins, pink arrows: Genes encoding hypothetical proteins, brown arrows: Mobile genetic element.

In these plasmids, the *mcr*-9 gene was located on an almost conserved region in the three *mcr-*9-harboring plasmids, of approximately 24,548 bp. *mcr*-9 was located adjacent to a cupin fold metalloprotein gene (*wbu*C) with the insertion sequences IS903 upstream and the IS26 downstream of both genes. Additionally, located upstream of this structure were genes associated with heavy metal metabolism (Figures 3, 4). No other AMR genes have been detected in this region.

The results obtained with BLAST analysis with the two previously published IncHI2 *mcr*-9-carrying plasmids revealed that our three IncHI2 *mcr*-9-carrying plasmids were closely related (88% coverage and 99.9% identity) to the IncHI2 ∼293 kb plasmid (p3846) described in Italy by Marchetti et al. (2021) in *E. cloacae*. As for the *mcr*-9/*bla*_*VIM*__–__1_-carrying IncHI2 plasmid (pRH-R27, size ∼299 kb) described in Germany in *S.* Infantis from a fattening pig farm, it shared with our three *mcr*-9-carrying plasmids a coverage range of 80–84% and an identity of 99.9%.

The specific region where *mcr*-9 was located in pMOL952, pMOL665, and pMOL500 was almost identical to the corresponding region of p3846 and pRH-R27 with an identity of 99% including the *wbu*C gene. The structural analysis revealed that pMOL952, pMOL665, and pMOL500 presented a mobile element (IS903) that was not identified in p3846 or pRH-R27 (Figures 3, 4).

The complete sequences of the four resolved plasmids were submitted to the European Nucleotide Archive^10^ under the project accession number PRJEB44718 (ERZ2110487, ERZ2110531, ERZ2110542, and ERZ2110516).

## Discussion

In this study, we report for the first time in Italy the presence and genomic features of 11 MDR, *mcr*-9-positive *S.* Typhimurium, and single-locus variant isolates from food-producing animals (fattening pigs and veal calves) belonging to the emerging ST34 clone, with some of them also displaying resistance to highest priority critically important antimicrobials (HPCIAs), as third- and fourth- generation cephalosporins. Indeed, plasmid-borne *mcr* genes pose a significant threat to public health at international level, not only because of the colistin-resistant phenotype they can induce but also because they can be transferred horizontally to foodborne pathogens in combination with other resistance genes, with the potential to be transmitted to humans and impair the treatment options.

From the Mash analysis, we identified 21 ST34 isolates within the same parental branch (both the 11 *mcr*-9 positive and the 10 negative ones) harboring large-size (∼277–297 kb) IncHI2/2A plasmids and a similar MDR gene pattern (Figure 2 and Supplementary Figure 1). Interestingly, only these isolates shared the presence of these plasmid types and presented a greater abundance of AMR genes compared to the rest of the *Salmonella* genomes analyzed. Because of the intrinsic characteristic of the Mash approach, the existence of a large-size IncHI2/2A plasmid harboring several similar AMR genes could have determined the high genetic relatedness of these isolates represented on the Mash tree.

Similarly to several previous findings (Carroll et al., 2019; Börjesson et al., 2020; Kananizadeh et al., 2020; Tyson et al., 2020), in our study, the presence of *mcr*-9 was not associated with clinical or microbiological resistance to colistin, and the *qse*C and *qse*B genes were absent in all the *mcr*-9-positive isolates detected. Indeed, the *mcr*-9 expression has been shown to be induced by the presence of colistin when this gene is located upstream of the two-component regulatory system *qse*BC. Therefore, *mcr*-9 may silently spread and remain undetected, unless induced by subinhibitory concentrations of colistin (Kieffer et al., 2019). However, the effectiveness of this regulatory system may be dependent on the genetic context (e.g., *mcr*-9 could be located on a plasmid or integrated into the chromosome) or may differ in relation to different strain backgrounds (e.g., serotype-dependent colistin susceptibility observed in *Salmonella* isolates) (Tyson et al., 2020), or other still unknown mechanisms could be involved. Additionally, the *mcr* gene family may undergo further genetic microevolution (and possibly to changes in the susceptibility pattern to colistin) due to selection pressure in the food-producing animal industry.

In our three selected isolates, *mcr*-9 was demonstrated to be located on IncHI2/IncHI2A plasmids by the hybrid (Illumina–Oxford Nanopore) approach, together with other AMR genes conferring resistance to different antimicrobial classes, including the *bla*_*SHV*__–__12_ ESBL gene type detected in the two plasmids of pig origin, and different genes involved in heavy metals metabolism. Similarly, previous findings detected IncHI2- or IncHI2A-type plasmids in *mcr*-9 positive Enterobacteriaceae, often together with other resistance determinants (including HPCIAs) and to heavy metals mainly from human patients (Carroll et al., 2019; Chavda et al., 2019; Kieffer et al., 2019; Bitar et al., 2020; Faccone et al., 2020; Kananizadeh et al., 2020; Lin et al., 2020; Osei Sekyere et al., 2020; Soliman et al., 2020; Tsui et al., 2020; Umeda et al., 2020; Elbediwi et al., 2021; Marchetti et al., 2021; Sun et al., 2021), and also from animal (Börjesson et al., 2020; Yuan et al., 2019; Borowiak et al., 2020; Haenni et al., 2020; Leite et al., 2020), food (Borowiak et al., 2020; Sadek et al., 2020; Tyson et al., 2020), and environmental (Kamathewatta et al., 2020) sources. Few reports also described *mcr*-9 integrated into the chromosome of *Citrobacter* (Ribeiro et al., 2021) and *Salmonella* (Pan et al., 2020; Tyson et al., 2020) isolates, with a genetic context similar to the structure observed in *mcr*-9-harboring plasmid sequences, suggesting a possible *mcr*-9 transfer as a gene cassette between plasmids and chromosomes (Pan et al., 2020).

However, only very few complete IncHI2 plasmid sequences carrying *mcr*-9 from food-producing animals and related foodstuff are available in public repositories for comparison, so far. In this regard, we fully reconstructed two *mcr*-9-positive IncHI2 plasmids from the monophasic variant of *S.* Typhimurium isolated from pig sources and one from the cecal content of a veal calf. To the best of the authors’ knowledge, this represents the first report of MDR, *mcr*-9-positive Enterobacteriaceae detected in bovine animals.

All 11 IncHI2 plasmids harboring *mcr*-9 were assigned to ST1 by pMLST analysis. IncHI2-ST1 plasmids have been the most frequently reported IncHI2 ST, representing a major vehicle in mediating *mcr*-9 and AMR gene dissemination also in Enterobacteriaceae isolates from clinical settings (Li et al., 2020). Interestingly, the *mcr*-9-negative IncHI2 plasmid we sequenced with ONT was assigned to another ST, ST4.

IncHI2 plasmids are known carriers of resistance determinants not only to antibiotics but also to heavy metals such as silver, mercury, arsenic, copper, tellurium, and others with the potential to coselect for the concomitant presence of *mcr*-9 (Tyson et al., 2020).

Moreover, pigs are considered one of the most significant vectors for the monophasic variant of *S.* Typhimurium ST34, and pork meat represents one of the main infection sources for humans (Biswas et al., 2019). As copper and zinc supplementation are commonly used in the swine industry (or in the case of therapeutical use of ZnO also to control postweaning diarrhea), heavy metals could accumulate in the environment, leading to a selection of ST34 isolates resistant to heavy metals and to IncHI2 plasmids frequently associated with various metal tolerance genes (Biswas et al., 2019).

IncHI2 plasmids have been reported to be diverse in terms of the overall genetic structure, while *mcr*-9 has been reported to be consistently located in the sil-cop region (Li et al., 2020). As previously described (Li et al., 2020), in this region, the core structure of all reported *mcr*-9 cassettes was rcnR-rcnA-pcoE-pcoS-IS903-mcr-9-wbuC, with the genetic content immediately upstream of *mcr-*9 mostly conserved. Differently, the gene content located downstream of *mcr*-9 was reported to be genetically diverse (silver resistance determinants and *qse*B-C regulators absent in most plasmids), and transposon elements were not identified.

In our plasmids, *mcr*-9 was located in a plasmid region (∼30 kb) lacking different genetic elements of the core structure, compared to the other plasmid sequences subjected to BLAST analysis (Figure 4). In particular, *mcr*-9 was flanked by the *wpu*C gene and the IS903 element with and additional IS element (IS26) located downstream *wpu*C, suggesting the potential ability to mobilize this gene. Heavy metal resistance genes were also identified (as arsenic and nickel) in the same region, but accordingly to previous findings, silver resistance determinants and *qse*B-C regulators were absent.

In conclusion, the spread of MDR *S.* Typhimurium, including monophasic ST34, has widely challenged the treatment options to control foodborne infections (Biswas et al., 2019). This can be of even more concern, especially when there is concomitant evidence of MDR genes against HPCIAs, such as colistin, extended-spectrum cephalosporins, and (fluoro)quinolones. Therefore, genomics and WGS-based surveillance are increasingly indispensable to achieve better insights also into the genetic environment and features of plasmid-mediated AMR and the relationships with bacterial pathogenic hosts, as in the case of such IncHI2 plasmids harboring *mcr*-9, that can be transferred horizontally also to major *Salmonella* serovars spreading along the food chain.

## Data Availability Statement

The datasets presented in this study can be found in online repositories. The names of the repository/repositories and accession number(s) can be found in the article/Supplementary Material.

## Author Contributions

AF, VC, PA, and ED conceived and designed the experiments. FF, PDM, MI, IM, AG, and ED performed the experiments. ED, PA, VC, AF, EC, and VD analyzed the data. VC, PA, ED, and AF wrote the manuscript. All authors contributed to manuscript revision, read, and approved the submitted version.

## Conflict of Interest

The authors declare that the research was conducted in the absence of any commercial or financial relationships that could be construed as a potential conflict of interest.

## Footnotes

1 https://eur-lex.europa.eu/legal-content/EN/TXT/HTML/?uri=CELEX:32013D0652&from=it

2 https://www.eurl-ar.eu/CustomerData/Files/Folders/23-eqas/558_2020-protocol-eurl-ec-salm-camp.pdf

3 https://github.com/tseemann/abricate

4 https://cge.cbs.dtu.dk/services/ResFinder/

5 https://cge.cbs.dtu.dk/services/PlasmidFinder/

6 https://github.com/tseemann/mlst

7 https://cge.cbs.dtu.dk/services/pMLST/

8 http://www.mgc.ac.cn/VFs/

9 https://www.eucast.org/

10 http://www.ebi.ac.uk/ena

## Supplementary Material

Supplementary Figure 1 **(A)** Presence or absence of plasmid replicons in the 177 *Salmonella* genomes identified by Mash analysis. **(B)** Presence or absence of AMR genes in the 177 *Salmonella* genomes identified by Mash analysis.

Supplementary Table 1 Metadata, phenotypic AMR patterns, and STs in 177 *S.* Typhimurium and monophasic variant isolates.
