# Supplementary figures and images for: Emergence of IncHI2 Plasmids With Mobilized Colistin Resistance (mcr)-9 Gene in ESBL-Producing, Multidrug-Resistant Salmonella Typhimurium and Its Monophasic Variant ST34 From Food-Producing Animals in Italy
Source: Front Microbiol. 2021 Jul 16;12:705230. doi: 10.3389/fmicb.2021.705230 (PMC8322855; doi:10.3389/fmicb.2021.705230)

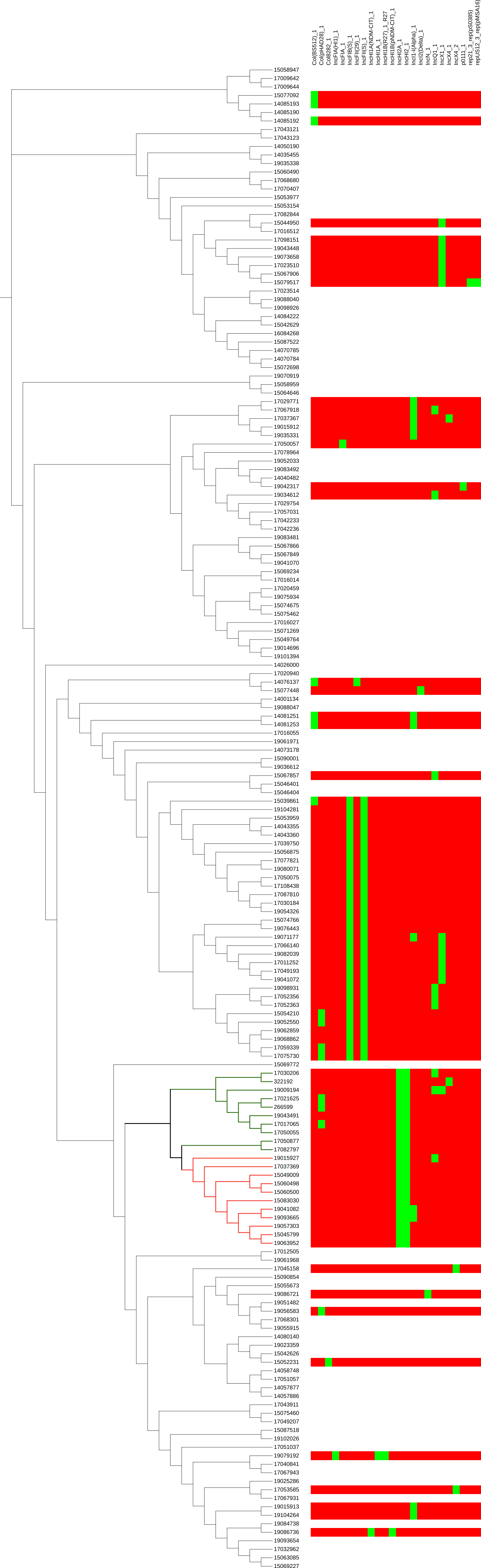

Supplement: Supplementary Figure 1 — (A) Presence or absence of plasmid replicons in the 177 Salmonella genomes identified by Mash analysis. (B) Presence or absence of AMR genes in the 177 Salmonella genomes identified by Mash analysis. [file Image_1.JPEG]
